# Ivarmacitinib in atopic dermatitis presenting with prurigo nodularis-like lesions and alopecia areata: a case report

**DOI:** 10.3389/fimmu.2026.1836241

**Published:** 2026-07-31

**Authors:** Xiaofan Liao, Yuan Lu

**Affiliations:** 1 Department of Dermatology, Affiliated Nanshan Hospital of Shenzhen University, Shenzhen, China; 2 Medical School, Shenzhen University, Shenzhen, China

**Keywords:** alopecia areata, atopic dermatitis, immune-mediated diseases, ivarmacitinib, Janus kinase 1 inhibitor, prurigo nodularis-like lesions

## Abstract

Atopic dermatitis (AD) is a chronic, relapsing inflammatory skin disorder characterized by intense pruritus, eczematous lesions, and significant impairment of quality of life. In severe or chronic cases, AD can present with diverse atypical phenotypes, among which prurigo nodularis (PN)-like lesions are clinically challenging due to their recalcitrance to conventional therapies. Patients with severe AD are also at an increased risk of comorbid immune-mediated diseases, such as alopecia areata (AA), a non-scarring hair loss disorder driven by aberrant immune responses. Ivarmacitinib, a highly selective oral Janus kinase 1 (JAK1) inhibitor, has been approved by the National Medical Products Administration (NMPA) of China for moderate-to-severe atopic dermatitis (April 2025) and alopecia areata (June 2025). Herein, we report a case of a 28-year-old male patient with a long-standing history of AD who developed generalized PN-like lesions and concurrent AA (with a 2-year disease duration) and androgenetic alopecia (AGA). Conventional therapies including topical corticosteroids and antihistamines failed to yield satisfactory outcomes. Following treatment with ivarmacitinib (8 mg once daily), the patient showed rapid improvements in skin lesions, pruritus, and hair regrowth. This case suggests that ivarmacitinib may be beneficial for complex AD phenotypes with comorbid immune-mediated hair loss.

## 1 Introduction

Atopic dermatitis (AD) is a heterogeneous inflammatory skin disease with a complex pathogenesis involving genetic predisposition, immune dysregulation, and environmental factors (1). Its clinical manifestations are highly variable, ranging from typical erythematous, scaly eczematous lesions to severe, treatment-resistant phenotypes such as erythrodermic AD and prurigo nodularis (PN)-like lesions (2). PN-like lesions in AD are characterized by hyperkeratotic, intensely pruritic nodules, which develop as a result of chronic scratching-induced neuroimmune dysregulation and excessive tissue repair responses (3). This phenotypic transformation significantly increases the therapeutic burden and further impairs patients’ quality of life.

Notably, severe AD is often associated with comorbid immune-mediated diseases, with alopecia areata (AA) being one of the most common (4). AA is an autoimmune disorder characterized by non-scarring hair loss, driven by the infiltration of autoreactive T cells and the overproduction of pro-inflammatory cytokines such as interleukin 15 (IL-15) and interferon γ (IFN-γ) (5). Emerging evidence also suggests a role for type 2 immune responses in the pathogenesis of AA, highlighting the complex interplay of immune pathways in this disease (6).

A key commonality between AD (including PN-like phenotypes) and AA is their reliance on the Janus kinase-signal transducer and activator of transcription (JAK-STAT) signaling pathway for cytokine-mediated inflammation (7). Cytokines critical to AD pathogenesis (e.g., IL-4, IL-13, IL-31) and AA (e.g., IFN-γ, IL-15) primarily signal through JAK1-dependent complexes (8, 9). This shared signaling axis provides a strong rationale for targeting JAK1 in patients with concurrent AD and AA.

Ivarmacitinib is a highly selective JAK1 inhibitor that exerts anti-inflammatory and immunomodulatory effects by reversibly binding to the ATP-binding site of JAK1, thereby blocking the activation of downstream STAT molecules (10). It has been shown to rapidly alleviate pruritus and improve skin lesions in patients with moderate-to-severe AD in phase 3 clinical trials (8). Furthermore, a phase 3 trial demonstrated that ivarmacitinib is effective for adults with severe alopecia areata (11). Here, we present a detailed case of a patient with AD presenting with PN-like lesions and concurrent AA/AGA who responded favorably to ivarmacitinib treatment, aiming to provide clinical evidence for the management of such complex cases.

## 2 Case presentation

A 28-year-old male patient was referred to our department with a long-standing history of AD, which had been diagnosed in childhood. Approximately 4 months prior to presentation, his skin condition acutely exacerbated, with the development of generalized, intensely pruritic hyperkeratotic nodules involving the trunk and extremities. Concomitantly, he had a 2-year history of progressive hair loss that had not been formally evaluated. The itching and hair loss greatly affected his sleep and had a significant impact on his social life and work. Before referral, he had been treated with topical corticosteroids and oral antihistamines continuously for 8weeks without adequate response. For hair loss, he had tried topical minoxidil 2% and oral finasteride for 6 months without great improvement. And he also received intramuscular compound betamethasone for 3 months also without noticeable benefit, and stopped treatment. Additionally, he also had a history of asthma and allergic rhinitis. The patient’s father has allergic rhinitis and allergic asthma; his mother has atopic dermatitis and urticaria. The clinical timeline is summarized in **Table 1**.

**Table 1:** Clinical timeline of the patient’s history, interventions, and outcomes.

Event	Time point
Diagnosis of atopic dermatitis (AD); history of asthma and allergic rhinitis	Childhood (more than 10 years ago)
Onset of progressive hair loss (diagnosed later as AA and AGA); diagnosis of Crohn’s disease (ileocolonic type, B1, moderate active phase)	2 years before presentation
Patient receives for alopecia: oral finasteride + topical minoxidil foam (6 months, no improvement); intramuscular compound betamethasone + topical minoxidil + halobetasol cream (3 months, no benefit); all treatments stopped	1.5 years before presentation
Initiation of adalimumab (40 mg subcutaneously every 2 weeks) for Crohn’s disease after failure of oral mesalazine	18 months before presentation
Crohn’s disease: clinical remission; fecal calprotectin normalizes	16 weeks after adalimumab start
Follow-up colonoscopy shows mucosal healing; adalimumab continued	48 weeks after adalimumab start
Acute exacerbation of AD: generalized prurigo nodularis (PN)-like lesions develop	2025.06
Presentation to our department; physical examination: EASI 22.5, NRS pruritus 7, DLQI 21, SALT 41.6; laboratory: IgE 1860 IU/mL, eosinophilia; skin biopsy confirms PN-like AD; trichoscopy and pull test confirm AA/AGA; ivarmacitinib 8 mg once daily initiated (no concomitant alopecia or topical AD therapies)	Baseline (2025.10)
Follow-up: EASI 10.6 (↓52.9%), NRS 4, SALT 31.3 (↓24.8%); mild acneiform eruption on back (resolves spontaneously)	Month 1 (2025.11)
Follow-up: EASI 5.4 (↓75.6%), NRS 2, SALT 20.5 (↓50.7%), DLQI 8; near-complete flattening of PN-like nodules; significant hair regrowth; Last adalimumab dose (discontinued due to sustained remission, as advised by gastroenterologist)	Month 2 (2025.12)
Maintain with Ivarmacitinib monotherapy alone; maintain stability of Crohn’s disease	After 7 months ongoing

**Figure 1:**
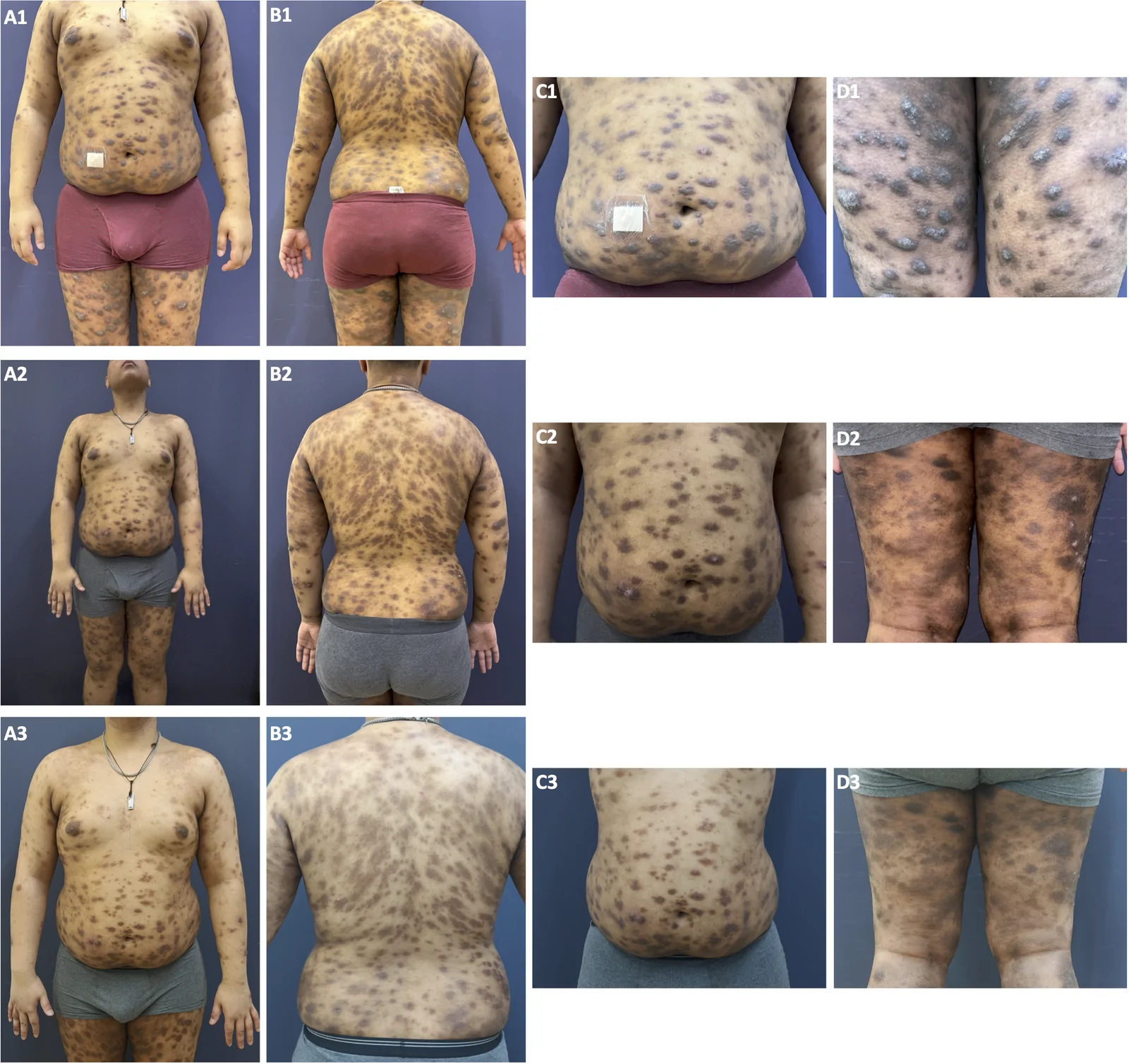
Clinical manifestations of skin lesions before and after ivarmacitinib treatment. Before treatment **(A1–D1)** Diffuse hyperpigmented patches and hyperkeratotic nodules with lichenification on the trunk and extremities (EASI = 22.5). After 1 month of treatment **(A2–D2)** Noticeable flattening of nodules, reduced erythema, and improved skin texture (EASI = 10.6). After 2 months of treatment **(A3–D3)** Almost complete flattening of nodules, significant lightening of hyperpigmentation, and marked improvement in skin lesions (EASI = 5.4).

**Figure 2:**
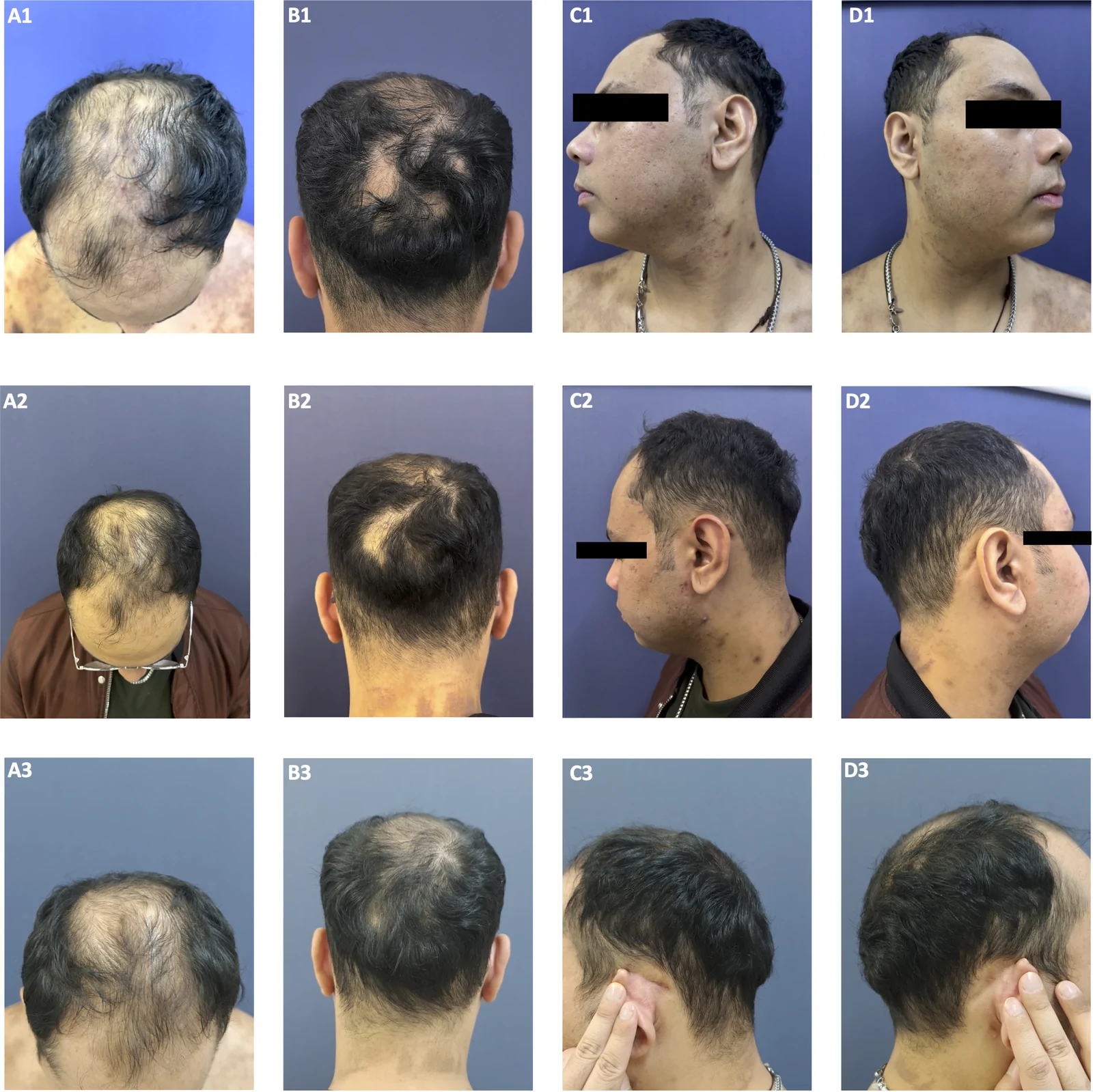
Clinical manifestations of alopecia before and after ivarmacitinib treatment. Before treatment **(A1–D1)** Diffuse hair thinning on the vertex and irregularÛald patches on the occipital and temporal regions (SALT = 41.6). After 1 month of treatment **(A2–D2)** Increased hair density on the vertex and egrowth in the left temporal region (SALT = 31.3). After 2 months of treatment **(A3–D3)** Significant reduction in bald areas and further improvement n hair density (SALT = 20.5).

**Figure 3:**
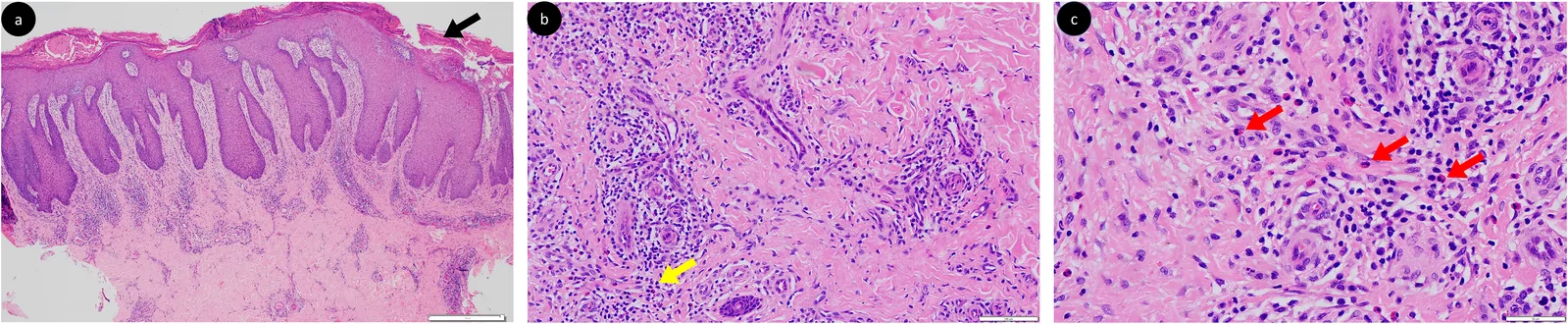
Histopathological findings of the abdominal skin lesion (Hematoxylin and eosin staining, **(a)** Marked epidermal thickening with hyperkeratosis and acanthosis (black arrow,×40), **(b)** Moderately dense perivascular and interstitial inflammatory infiltrate in the superficial and mid-dermis (yellow arrow,×200). **(c)** the inflammatory infiltrate composed predominantly of lymphocytes, with admixed scattered eosinophils (red arrow, ×400).

Physical examination revealed diffuse hyperpigmented patches and hyperkeratotic nodules over the trunk, upper limbs, and lower limbs. Most nodules were markedly lichenified, with crusting observed on some lesions (**Figures 1A1–D1**). The Eczema Area and Severity Index (EASI) score was 22.5, indicating severe AD, the Numerical Rating Scale (NRS) for pruritus was 7 (on a scale of 0-10, with 10 representing the most severe pruritus), and the Dermatology Life Quality Index (DLQI) was 21, reflecting severe impairment of quality of life. Regarding hair, diffuse hair thinning was noted on the vertex of the scalp, with irregularly sized bald patches on the occipital and temporal regions (**Figures 2A1, D1**). Through trichoscopy and a positive pull test at active margins, he was diagnosed as alopecia areata (AA) and androgenetic alopecia (AGA). The Severity of Alopecia Tool (SALT) score was 41.6, consistent with moderate-to-severe alopecia. Laboratory examinations showed a markedly elevated serum immunoglobulin E (1860 IU/mL; normal <100 IU/mL) and a mild eosinophil count (0.65×10^9^/L; normal range: 0.02-0.52×10^9^/L). Liver and kidney function, electrolyte levels, and infectious disease screening (including hepatitis B, hepatitis C, HIV, and syphilis) were all within normal limits. A pathological biopsy of the abdominal nodule revealed epidermal thickening with hyperkeratosis, acanthosis, and superficial dermal inflammatory infiltrate consisting of lymphocytes and eosinophils (**Figure 3**). Differential diagnoses considered included classic prurigo nodularis (usually limited to extensor surfaces without eczematous background), hypertrophic lichen planus, nodular scabies, cutaneous lymphoma, and perforating disorders, all of which were ruled out based on clinical and histopathological features. The diagnosis of PN-like AD was confirmed using Hanifin-Rajka criteria (12), combined with generalized hyperkeratotic nodules, histopathology, elevated IgE, and personal atopic history.

The patient was diagnosed with Crohn’s disease (ileocolonic type, B1, moderate active phase). Initial treatment with oral mesalazine was ineffective, and therapy was escalated to subcutaneous adalimumab. After 16 weeks of adalimumab treatment, clinical symptoms resolved and fecal calprotectin normalized. Maintenance therapy continued to week 48, when follow-up colonoscopy demonstrated mucosal healing. Adalimumab was continued thereafter.

Given the extensive and severe skin lesions, intense pruritus, refractory nature to conventional therapies (topical corticosteroids and antihistamines), and comorbid AA, the decision to continue adalimumab during the initial two months of ivarmacitinib therapy was made jointly by the dermatology and gastroenterology teams. The dosage was determined based on the patient’s weight (85 kg) as 8 mg once daily while IBD remained stable during treatment with ivarmacitinib and given that the patient’s Crohn’s disease had been in sustained remission on adalimumab, the gastroenterologist advised against immediate withdrawal to avoid potential IBD flare during the introduction of a new systemic immunomodulator. Regular follow-up was scheduled at 1 month and 2 months after treatment initiation to assess efficacy and safety. As the patient remained in sustained clinical and endoscopic remission. During ivarmacitinib treatment for 2 months, the patient’s Crohn’s disease remained stable, with no symptoms or biomarker elevation. His gastroenterologist advised him to stop using adalimumab. He is still under follow-up and Crohn’s disease remains stable. During the entire follow-up period, the patient used only bland emollients; no topical corticosteroids, oral antihistamines, minoxidil, or intralesional corticosteroid injections were permitted or used. The patient is a Hong Kong resident whose Crohn’s disease had been managed with long-term adalimumab in Hong Kong. During adalimumab treatment, the patient’s alopecia areata and AD-related PN-like lesions showed no improvement and progressively worsened, suggesting that adalimumab was ineffective for these conditions. After ivarmacitinib initiation, the Hong Kong gastroenterologist was informed and discontinued adalimumab at month 2. At month 4, colonoscopy confirmed that the patient’s Crohn’s disease remained stable on ivarmacitinib monotherapy. This combined approach was off-label for both agents and was undertaken after thorough discussion of the potential risks (including infection, thromboembolic events, and malignancy) and benefits with the patient, who provided informed consent.

### 2.1 Follow-up and outcomes

#### 2.1.1 Efficacy outcomes

1 month after treatment initiation: The patient reported a significant reduction in pruritus (NRS score decreased from 7 to 4). Physical examination showed noticeable flattening of hyperkeratotic nodules, reduced erythema of background plaques, and improved skin texture, particularly on the abdomen and lower limbs (**Figures 1A2–D2**). The EASI score decreased from 22.5 to 10.6 (a 52.9% reduction). For hair loss, increased hair density was observed on the vertex, with notable regrowth in the left temporal region (**Figures 2A2–D2**). The SALT score decreased to 31.3 (a 24.8% reduction from baseline).

**Figure 4:**
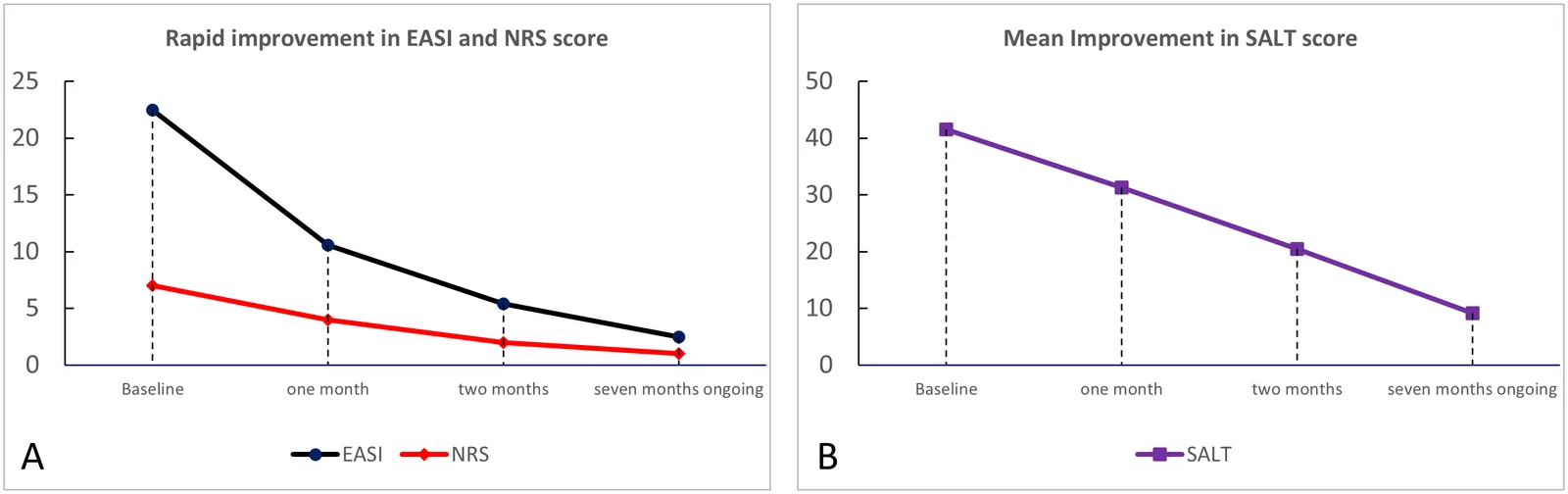
Temporal trends of efficacy parameters during ivarmacitinib treatment. **(A)** EASI score and NRS score for pruritus showed continuous reduction; **(B)** SALT score for alopecia demonstrated significant improvement. All parameters improved rapidly within 8 weeks.

2 months after treatment initiation: Pruritus was nearly completely relieved (NRS score decreased to 2). Most hyperkeratotic nodules had completely flattened, and hyperpigmentation had significantly lightened (**Figures 1A3–D3**). The EASI score further decreased to 5.4 (a 75.6% reduction from baseline). Regarding alopecia, the bald area on the occipital region was reduced by 50% compared with baseline, and the left temporal bald area was almost completely resolved (**Figures 2A3, D3**). The SALT score improved to 20.5 (a 50.7% reduction from baseline). The DLQI score decreased to 8, indicating a significant improvement in quality of life (**Figure 4**).

#### 2.1.2 Safety outcomes

**Figure 5:**
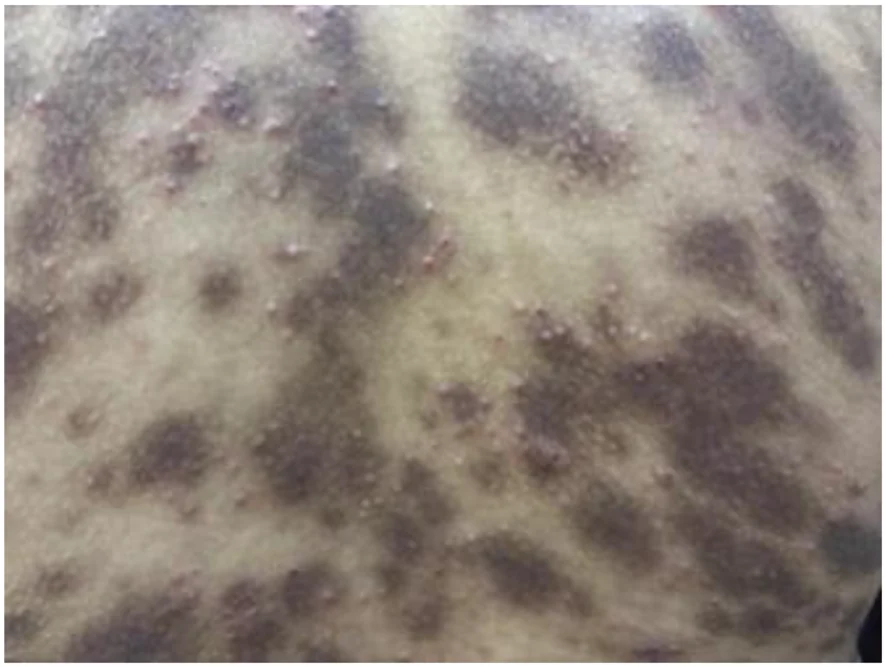
Adverse event: Mild acneiform eruption on the back 1 month after ivarmacitinib treatment, which resolved spontaneously without intervention.

During the 2-month treatment period, the patient developed a mild, self-limiting acneiform eruption on the back (**Figure 5**) 1 month after treatment initiation. No other adverse events such as fever, fatigue, gastrointestinal discomfort, or abnormalities in liver/kidney function were observed. The acneiform eruption resolved spontaneously within 2 weeks without any intervention and was considered a JAK1 inhibitor-related adverse event.

## 3 Discussion

This case describes a complex clinical scenario involving a special phenotype of AD (PN-like lesions) complicated by AA and AGA, which responded effectively to the selective JAK1 inhibitor ivarmacitinib. The concurrent improvement in both skin and hair follicle pathology highlights the shared immunopathological mechanisms underlying these conditions and the therapeutic potential of JAK1 targeting in complex immune-mediated diseases.

### 3.1 Pathophysiological insights into the complex phenotype

#### 3.1.1 PN-like AD: a severe endotype of AD

The patient’s transformation from chronic AD to generalized PN-like lesions reflects a severe endotype driven by persistent inflammation and the “itch-scratch cycle”. Unlike classic PN, which typically localizes to the extensor surfaces of the limbs (13), this patient’s widespread involvement suggests a systemic escalation of the inflammatory process. The pathogenesis of PN-like AD is dominated by type 2 immune responses, with key cytokines including IL-4, IL-13, and IL-31 (3). IL-31, in particular, is a major mediator of pruritus, and it signaling via JAK1/JAK2 complexes drives the intense itching that perpetuates the scratch cycle (14). Chronic scratching further induces epidermal hyperplasia, fibrosis, and the formation of hyperkeratotic nodules, creating a vicious cycle of inflammation and tissue remodeling.

#### 3.1.2 Comorbid AA: overlapping immune pathways

AA is characterized by the breakdown of immune privilege in hair follicles, mediated by autoreactive T cells and pro-inflammatory cytokines (5). While Th1-type cytokines (IFN-γ, IL-15) have long been recognized as central driver (15), recent studies have highlighted the involvement of Th2-type responses in some patient (6). Notably, both Th1 and Th2 cytokines involved in AA signal through JAK1-dependent pathways (9), providing a mechanistic link between AA and AD. The coexistence of these two diseases in our patient underscores the complexity of immune dysregulation in atopic individuals and the need for therapies targeting shared signaling pathways.

### 3.2 Rationale for JAK1 inhibition with ivarmacitinib

Conventional therapies for severe AD (e.g., topical corticosteroids, systemic immunosuppressants) and AA (e.g., topical minoxidil, corticosteroid injections) often fail to address the underlying immune dysregulation, particularly in complex phenotypes. Biologics targeting single cytokines (e.g., anti-IL-4/13, anti-IL-31) may have limited efficacy in patients with overlapping endotypes, as they only inhibit one arm of the inflammatory response (16). In contrast, JAK1 inhibitors act as “upstream regulators” that simultaneously block multiple cytokine pathways involved in both diseases. While TNF inhibitors are a standard therapy for IBD, JAK inhibitors may also be effective by simultaneously suppressing multiple pro-inflammatory cytokine signals that converge on the JAK–STAT pathway, a key axis in intestinal immune dysregulation and impaired mucosal homeostasis. In the AMBER2 phase II trial, ivarmacitinib demonstrated superior efficacy and higher remission rates versus placebo in moderate-to-severe active ulcerative colitis, with a favorable tolerability profile (17). At the time of ivarmacitinib initiation, the patient was still receiving adalimumab (40 mg every 2 weeks) for Crohn’s disease, which had been in sustained remission. Ivarmacitinib and adalimumab were used concurrently for the first 2 months. After the 2-month follow-up, adalimumab was discontinued as advised by his gastroenterologist, while ivarmacitinib was continued. During the entire 2-month period, the patient’s Crohn’s disease remained stable with no symptoms or biomarker elevation.

Ivarmacitinib’s high selectivity for JAK1 allows it to specifically target the signaling of IL-4, IL-13, and IL-31 (critical for AD and pruritus) while also inhibiting IFN-γ and IL-15 (key for AA) (8, 10). By blocking JAK1, ivarmacitinib breaks the itch-scratch cycle in AD, reduces skin inflammation and fibrosis, and promotes the resolution of PN-like nodules. Simultaneously, it suppresses the autoimmune attack on hair follicles in AA, restoring immune privilege and facilitating hair regrowth. This dual mechanism of action explains the concurrent improvement in both skin lesions and alopecia observed in our patient.

It is important to acknowledge that the patient received concurrent adalimumab and ivarmacitinib for the first two months. While the observed cutaneous and hair improvements occurred during this combination period, adalimumab alone had not previously been associated with hair regrowth in this patient, and his AD had been poorly controlled despite prior topical therapies. Nevertheless, the concurrent use of two systemic immunomodulators represents a significant confounder, and the contribution of each agent to the clinical outcomes cannot be definitively distinguished. Notably, the patient’s alopecia and AD lesions had progressively worsened despite long-term adalimumab therapy, and improved only after ivarmacitinib introduction. This temporal patternmay supports the attribution of clinical improvement to ivarmacitinib rather than to adalimumab. Furthermore, emerging evidence supports the efficacy of JAK1 inhibitors in inflammatory bowel disease (18); in this patient, adalimumab was successfully withdrawn after two months of combination therapy, and Crohn’s disease remained stable on ivarmacitinib monotherapy, consistent with the known therapeutic potential of JAK inhibition in IBD. The combination of a JAK1 inhibitor and a TNF inhibitor is not currently recommended in product labeling due to the lack of safety data and the potential for additive immunosuppression. In this case, the decision was individualized based on multidisciplinary input and close monitoring, but this approach should not be generalized without further evidence.

### 3.3 Clinical implications and limitations

This case provides clinical evidence for the use of ivarmacitinib in the treatment of complex, refractory phenotypes of AD combined with comorbid immune-mediated hair loss. The rapid and significant improvements in EASI, NRS, and SALT scores, along with a favorable safety profile, suggest that ivarmacitinib may be a valuable therapeutic option for such patients. However, several limitations should be noted: (1) This is a single-case report, and larger controlled studies are needed to confirm the generalizability of these findings; (2) The follow-up period was relatively short (2 months), and long-term efficacy and safety (e.g., risk of infections, hematological abnormalities) require further monitoring.

The concurrent use of adalimumab and ivarmacitinib for the first two months is a major confounder that limits attribution of the observed improvements solely to ivarmacitinib. Although adalimumab had been ineffective for the patient’s AD and alopecia prior to ivarmacitinib initiation, its immunomodulatory effects cannot be entirely excluded. Furthermore, the safety of combining JAK1 and TNF inhibitors is not well established, and this strategy carries potential additive risks of infection, malignancy, and thromboembolic events. This combination was used off-label and should not be considered routine practice. We also acknowledge the challenge of cross-border care coordination, as the patient’s IBD was managed by a different gastroenterologist in Hong Kong, and direct multidisciplinary communication was not feasible due to practice constraints. Nevertheless, the Hong Kong gastroenterologist was informed of the treatment and subsequently withdrew adalimumab after confirming disease stability on ivarmacitinib.

## 4 Conclusion

In conclusion, this case report provides preliminary, hypothesis-generating evidence that the selective JAK1 inhibitor ivarmacitinib may improve skin lesions, pruritus, and hair regrowth in a patient with severe AD presenting with PN-like lesions and comorbid AA/AGA. However, given the short follow-up (2 months), lack of trichoscopy, non-standardized photography, potential confounding from concurrent adalimumab, and the inherent limitations of a single case report, these findings should be interpreted cautiously. Ivarmacitinib represents a potential targeted option for such complex immune-mediated phenotypes, but larger, prospective, long-term studies are necessary to confirm efficacy, establish safety profiles, and define optimal patient selection. The findings presented here are hypothesis-generating and require validation in controlled clinical setting.

## Data Availability

The original contributions presented in the study are included in the article/supplementary material. Further inquiries can be directed to the corresponding author.
