# Correction: Ivarmacitinib in atopic dermatitis presenting with prurigo nodularis-like lesions and alopecia areata: a case report

**DOI:** 10.3389/fimmu.2026.1961352

**Published:** 2026-08-18

**Authors:** Xiaofan Liao, Yuan Lu

**Affiliations:** 1 Department of Dermatology, Affiliated Nanshan Hospital of Shenzhen University, Shenzhen, China; 2 Medical School, Shenzhen University, Shenzhen, China

**Keywords:** alopecia areata, atopic dermatitis, immune-mediated diseases, ivarmacitinib, Janus kinase 1 inhibitor, prurigo nodularis-like lesions

There was a mistake in the caption of **Figure 3** as published. The description for **Figure 2** was mistakenly placed in the position of **Figure 3**, resulting in the descriptions of **Figure 2** and **3** being duplicates. The corrected caption of **Figure 3** appears below.

“Histopathological findings of the abdominal skin lesion (Hematoxylin and eosin staining, **(a)** Marked epidermal thickening with hyperkeratosis and acanthosis (black arrow,×40), **(b)** Moderately dense perivascular and interstitial inflammatory infiltrate in the superficial and mid-dermis (yellow arrow,×200). **(c)** the inflammatory infiltrate composed predominantly of lymphocytes, with admixed scattered eosinophils (red arrow, ×400).”

The original version of this article has been updated.

